# Efficacy of high-intensity home mechanical stretch therapy for treatment of shoulder stiffness: a retrospective review

**DOI:** 10.1186/s13018-022-03325-9

**Published:** 2022-09-29

**Authors:** Shaun Stinton, Samantha Beckley, Alicia Salamani, Devinne Dietz, Thomas Branch

**Affiliations:** 1 ArthroResearch LLC, 441 Armour Place NE, Atlanta, GA 30324 USA; 2 Ermi LLC, 2872 Woodcock Blvd. Suite 100, Atlanta, GA 30341 USA

**Keywords:** Shoulder, Adhesive capsulitis, Motion loss, Stiffness, Mechanical therapy, Non-operative treatment

## Abstract

**Background:**

Shoulder stiffness resulting in motion loss can be caused by numerous conditions, the most common of which is adhesive capsulitis. Surgical intervention is often necessary when conservative methods fail. High-intensity stretch (HIS) treatment may be able to provide increased motion gains while avoiding the cost and complications of surgery.

**Objectives:**

The purpose of this study was to review data from patients who were prescribed a HIS device to recover their shoulder motion to determine the efficacy of the device. The hypotheses were that patients would achieve significant range of motion (ROM) gains and that ROM would increase to a level at which patients would be able to avoid a motion loss surgery and perform activities of daily living.

**Methods:**

Clinical notes were reviewed for patients whose progress plateaued after 4 weeks of therapy and were subsequently prescribed the HIS device after failing to meet their treatment goals. ROM data were recorded for external rotation, abduction, forward flexion, and internal rotation. Pre- and post-treatment ROM data were compared using *t*-tests.

**Results:**

Significant ROM gains were seen in all planes of motion (*p* < 0.001). Patients gained an average of 29.9° in external rotation with a last recorded rotation of 59.2°. In abduction, patients gained 40.5° with a last recorded abduction of 123.3°. In forward flexion, patients gained 30.3° with a last recorded flexion of 138.7°. In internal rotation, patients gained 15.2° with a last recorded rotation of 57.6°. These last recorded ranges of motion were sufficient to perform nearly all activities of daily living.

**Conclusions:**

The HIS device was effective in treating patients with shoulder motion loss as demonstrated by the significant ROM gains in all planes of motion. The ability for a patient to recover lost motion quickly without surgery is of great value to quality of life and in healthcare cost savings. We believe this high-intensity stretch device should be considered for use by patients who are at risk for a motion loss surgery.

## Background

Shoulder stiffness leading to motion loss can be caused by several conditions. Adhesive capsulitis results from a number of etiologies and is one of the most common causes of motion loss in the shoulder. The condition can develop either gradually and without a known cause (primary) or after trauma or surgery (secondary) [1–4]. The term frozen shoulder is often used interchangeably with adhesive capsulitis and has the same ICD10 code. There are several other conditions with similar symptomatology (stiffness and pain) but differing ICD10 codes including ankylosis (bone-related), contracture (soft tissue related), internal joint derangement, etc. Up to 5% of Americans develop adhesive capsulitis with a peak incidence in persons between 40 and 70 years old [5–7]. Adhesive capsulitis is characterized by inflammation and stiffness in the shoulder capsule, the surrounding tissues, and in the glenohumeral joint. The dense collagen matrix that forms in the shoulder as a result of inflammatory response and subsequent fibrotic response leads to contracture of the capsuloligamentous complex [8]. Capsular stiffness and inflammation often result in chronic pain, which can lead to difficulty sleeping, difficulty concentrating, and depression. Contracture of the soft tissues in the shoulder leads to decreased volume within the joint and reduced range of motion (ROM) [9, 10] This decrease in motion can last for months or years in some patients while severely limiting day-to-day activities over that time. This delayed recovery can also prevent a timely return to work.

The majority of adhesive capsulitis patients achieve a good clinical outcome within 2 years and recover to a functional level through conservative treatment including steroid injections, nonsteroidal anti-inflammatory drugs, and physical therapy [11, 12]. However, up to 40% of patients suffer from continued severe motion loss, pain, and loss of function for longer than 3 years with up to 15% having a permanent disability [13, 14]. In these patients who have not responded to traditional conservative treatment alone, a critical decision point is reached. Historically there has been a choice between two options: (1) continued prolonged physical therapy or (2) a surgery such as manipulation under anesthesia (MUA) with or without an arthroscopic lysis of adhesions followed by additional physical therapy, doctor visits, and medications [15]. Any surgery can lead to significant downstream costs while also restarting the timeline for recovery. The direct costs associated with treating adhesive capsulitis in 2000 were $7 billion in the United States alone which would be the equivalent of $11.6 billion in 2022 [16]. Indirect costs related to missed work have not been reported to our knowledge, but could be even greater. Having an effective non-surgical treatment for adhesive capsulitis patients who do not recover through traditional conservative treatment as a third clinical option could lead to significant direct and indirect cost savings. These savings come from prevention of additional surgical intervention and the subsequent therapy and recovery time and costs which allows the patient to return to work sooner.

A third clinical option for recovery of lost motion that could help avoid prolonged physical therapy and secondary surgery is high-intensity home mechanical stretch (HIS) therapy. In comparison to low load prolonged stretch devices or static progressive stretch devices, this HIS therapy applies a higher force to the joint, allowing for shorter treatment times than the lower intensity devices. The HIS device investigated in the current study is hydraulically driven and is completely controlled by the patient. It allows the patient to stretch their shoulder up to the level applied during in-clinic physical therapy, but on a daily basis in the comfort of their own home. The goal of using the HIS device is to maximize total end-range time (TERT) of stretching at the end-range of motion of the joint. The TERT dose is a product of intensity, frequency and duration of passive stretching [17]. Following the TERT formula, HIS can permanently remodel contracted tissue in the shoulder. This HIS device has been previously shown to successfully treat motion loss in the shoulder [18, 19].

In one study, treatment outcomes (forward flexion and combined internal/external rotation) in 60 patients with postoperative adhesive capsulitis were compared between two groups: (1) patients who used the HIS device after reaching a plateau in their recovery following traditional physical therapy (PT + HIS) and (2) patients who showed no plateau in their recovery with physical therapy alone (PT only) [18]. Patients in the PT + HIS group had significantly worse initial elevation and combined rotation when compared to PT-only patients. Final elevation was statistically equivalent between the groups and combined rotation was significantly greater in PT + HIS patients. Gains in elevation and rotation were significantly greater for the PT + HIS patients than PT-only patients, with equivalent treatment times. These results showed that patients who were worse off initially were able to jump-start their recovery using the HIS device to achieve equivalent or greater results as compared to patients in the control group in the same amount of time.

In another study, treatment of motion loss due to adhesive capsulitis using the same HIS device was shown to be safe and effective in 36 frozen shoulder patients [19]. Patients were separated into groups based on the level of irritability (low or moderate/high) in their treated shoulder. There were no significant differences between the groups in pre-treatment ROM or post-treatment ROM in the injured shoulder or the uninvolved shoulder. In addition, there were no significant differences in post-treatment ROM between the treated shoulder and the uninvolved shoulder in either group. Ninety-seven percent of patients were able to avoid additional surgery. These results demonstrate that the HIS device is effective in restoring ROM and preventing a secondary surgery regardless of the irritability level of the shoulder. While the results from previous studies on this HIS device were promising, larger studies would further validate the clinical benefit and potential cost savings seen through using HIS therapy.

The purpose of this retrospective study was to review data from patients who were prescribed a HIS device to recover their shoulder motion to determine treatment efficacy. The primary hypothesis of the study was that patients treated with the device would achieve significant ROM gains in all planes of motion in the shoulder. A secondary hypothesis was that these ROM gains would be sufficient for patients to avoid a motion loss surgery and to perform activities of daily living.

## Methods

**Fig. 1:**
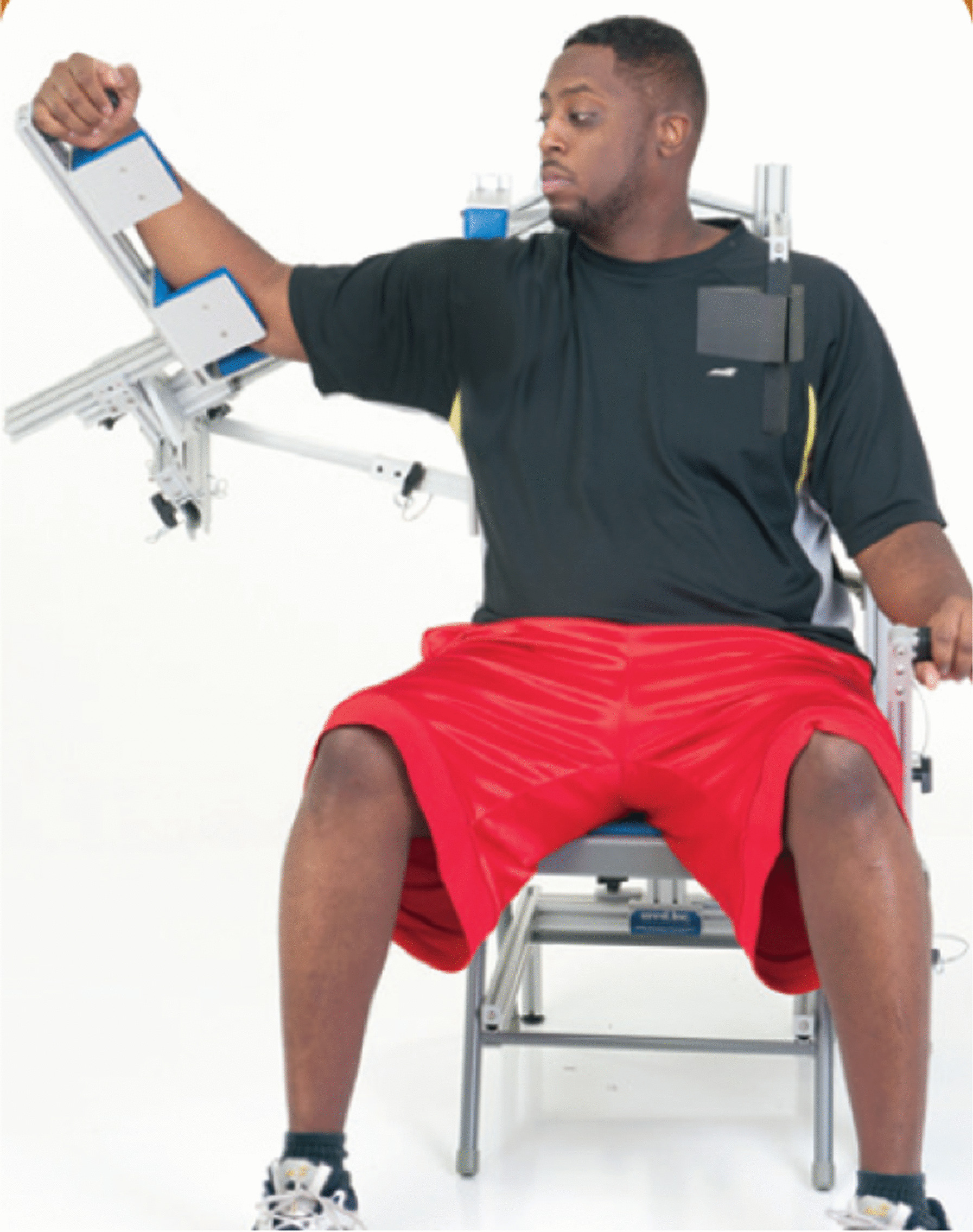
The Ermi Shoulder Flexionater set up to stretch the shoulder in combined abduction and external rotation. The device can also stretch external rotation or abduction individually (not pictured)

This study was exempt from institutional review board oversight and a waiver of authorization was granted prior to initiating the study. Records related to providing a HIS device (the Ermi Shoulder Flexionater—Fig. 1) were reviewed for all patients who were prescribed the device between January 1, 2010 and December 31, 2015. Patients were being treated due to a variety of conditions including frozen shoulder, rotator cuff repair, impingement syndrome, biceps tenodesis, superior labral tear from anterior to posterior (SLAP), chondroplasty, microfracture surgery, lysis of adhesions, acromioclavicular joint sprain, distal clavicle excision, subacromial decompression, humerus fracture, mastectomy, and shoulder replacement surgery.

The data collected included: (1) initial ROM; (2) last recorded ROM; (3) the number of days between those measurements; and (4) the number of days between device delivery and the last recorded ROM. The initial recorded ROM was the measured ROM that was taken closest to the time of delivery of the Shoulder Flexionater. Another set of ROM measurements was required when a re-certification of medical necessity occurred (generally on a monthly basis). The last recorded ROM measurement was the most recent measurement available in the physical therapy notes which was generally from the beginning of the last re-certification period. This measurement is most likely not the patient’s final ROM after completing treatment. The inclusion criteria for patient data were: (1) The patient must have 2 sets of ROM measurements for at least one plane of shoulder motion; (2) the initial ROM measurement must be taken within 30 days of device delivery (could be up to 30 days before delivery or 30 days after delivery); (3) the second ROM measurement must be at least 10 days post-device delivery; and (4) there must be at least 14 days between measurements. As an example, a patient could have their initial measurement 4 days before device delivery and their last measurement at 10 days after delivery.

The HIS device was prescribed when patients reached a plateau in their recovery after at least 4 weeks of physical therapy and were unable to meet their treatment goals [20]. These goals were based on injury type, surgical procedure, contralateral shoulder ROM, age and sex of the patient, and preoperative ROM. The device could be configured to stretch in external rotation, abduction, or internal rotation (Fig. 1). A representative of the company set up the device and trained the patient on its use at the time of delivery. The patient never changed the plane of stretch on their own. Generally, the device was setup to stretch in external rotation for approximately one month of treatment and then switched to stretch in abduction by the representative of the company. These are the two planes in which motion is most commonly lost in adhesive capsulitis patients [21]. In some patients, internal rotation was stretched in a subsequent month. In some cases, this protocol was different at the direction of the treating clinician. Patients used the device to stretch their shoulder during three sessions per day. In each session, the patient stretched the shoulder up to the maximum tolerable stretch (end-range of motion) for 10 min and then released the stretch for 10 min. This was followed by another 10 min period of end-range stretch. The patient can apply a torque in small increments of up to 125 Nm as shown in laboratory testing. Patients were instructed to stretch to the point of discomfort but not the point of pain. Therefore, patients would generally apply a torque well under the maximum level that the device can generate. The hydraulic nature of the device allowed for fine control of the stretch by the patient while allowing the patient to feel feedback as the stretch was advanced. The completely patient-controlled nature of the stretch allowed the patient to push to the end range of motion without the fear and muscle guarding that can occur when the stretch is applied by a physical therapist.

Records from 15,133 patients were reviewed. The ROM measurements taken using a goniometer were recorded from physical therapy progress notes. All measurements in a single patient were from the same clinic. A patient could have measurements from one or more planes of shoulder motion: external rotation, abduction, forward flexion, and internal rotation. Available measurements in the clinical notes were passive ROM in the majority of patients, but in rare cases (< 5%) only a complete set of active ROM measurements or a mix of active and passive measurements was available. In mixed cases, the data were only used if the initial measurement was a passive measurement. Since passive ROM is generally larger than active ROM, the patient’s improvement in ROM would most likely be underestimated, and not overestimated. Two or more ROM measurements that met the inclusion criteria for at least one plane of shoulder motion were available for 1871 patients. There were 1727 patients with data for external rotation, 1443 patients with data for abduction, 851 patients with data for forward flexion, and 457 patients with data for internal rotation. Patient populations were different between motions because available recorded measurements varied from patient to patient. Patients who did not have 2 sets of measurements for at least one plane of motion were excluded from the analysis. Measurements for forward flexion were included in the study even though the device did not stretch in that plane of motion because it is an important indicator of successful recovery.

Pre- and post-treatment ROM for external rotation, abduction, forward flexion, and internal rotation were compared using two-sample, equal variance *t* tests after *F* tests ensured equal variances. One-way analysis of variance was performed when comparing ROM measurements between age groups.

## Results

Pre- vs. post-treatment comparisons showed significant improvement from the initial measurement to the final measurement in all ranges of motion (*p* < 0.001 for all motions). For the 1871 total patients, the average days between device delivery and the final available ROM measurement was 69.0 days. The initial ROM, last recorded ROM, and ROM gain for each plane of shoulder motion are shown in Table 1.

**Table 1:** Range of motion data for each plane of shoulder motion

	External rotation	Abduction	Forward flexion	Internal rotation
*n*	1727	1443	851	457
Initial range of motion (°)	29.3 (19.5)	82.8 (32.0)	108.4 (34.1)	42.4 (21.3)
Last recorded range of motion (°)	59.2 (20.6)	123.3 (32.7)	138.7 (28.2)	57.6 (18.9)
Increase in range of motion (°)	29**.**9 (23.5)	40.5 (36.6)	30.3 (33.9)	15.2 (23.6)
*p* value	< 0.001	< 0.001	< 0.001	< 0.001

Data are mean (SD)

Table 2 shows treatment efficacy categorized by initial range of motion of the patient. Motion gains were greatest for patients with lower initial ROM measurements. Gains decreased as starting ROM increased. In patients that were in the highest category of starting ROM in each plane of motion (≥ 45° for external and internal rotation, ≥ 120° for abduction, and ≥ 130° for forward flexion) the gains were smaller (between 2.8° and 13.0°). These patients were likely being treated for lost motion in other planes and did not need significant gains in that specific plane of motion.

**Table 2:** Treatment efficacy categorized by starting range of motion

External rotation	< 15°	15–29°	30–44°	≥ 45°
*n*	384	464	461	418
Initial external rotation (°)	4.6 (6.5)	20.6 (3.8)	34.6 (4.5)	55.7 (10.9)
Last recorded external rotation (°)	53.2 (22.1)	54.5 (20.3)	60.1 (18.5)	68.7 (17.9)
External rotation gain (°)	48.6	33.9	25.5	13.0

Abduction	< 80°	80–99°	100–119°	≥ 120°
*n*	603	423	213	204
Initial abduction (°)	53.3 (18.2)	87.5 (5.1)	107.0 (5.8)	135.2 (148)
Last recorded abduction (°)	111.8 (33.4)	122.9 (29.6)	133.9 (26.7)	146.8 (25.8)
Abduction gain (°)	58.5	35.4	26.9	11.6

Forward flexion	< 80°	80–109°	110–129°	≥ 130°
*n*	138	249	191	273
Initial flexion (°)	52.2 (19.0)	92.2 (7.6)	117.8 (5.4)	145.0 (11.6)
Last recorded flexion (°)	120.9 (33.3)	130.7 (28.7)	142.1 (22.2)	152.7 (20.0)
Flexion gain (°)	68.7	38.5	24.3	7.7

Internal rotation	< 15°	15–29°	30–44°	≥ 45°
*n*	50	55	121	231
Initial internal rotation (°)	4.1 (4.9)	21.2 (4.1)	35.6 (4.6)	59.4 (12.0)
Last recorded internal rotation (°)	50.7 (19.3)	52.3 (25.4)	54.9 (16.5)	62.2 (16.9)
Internal rotation gain (°)	46.6	31.1	19.3	2.8

Data are mean (SD)

Guidelines for motion restrictions that would require a manipulation of the shoulder (after unsuccessful conservative treatment through physical therapy and injections) have been previously reported as a 30 degree restriction of passive motion in external rotation and at least one second plane of movement with 30 degree restriction compared to the contralateral shoulder [22]. In this study, we do not have ROM measurements for the contralateral shoulder, but we can use the ROM requirements to perform activities of daily living to define threshold values that would likely allow a patient to avoid a manipulation. Activities of daily living require 59° of external rotation and 121° of both abduction and forward flexion [23, 24]. If the threshold to avoid a manipulation is set 30° below the level required for activities of daily living, then 30° of external rotation and 90° of abduction and forward flexion would avoid a manipulation. The percentage of HIS patients in the lowest starting ROM categories (< 15° for external rotation and < 80° for abduction and forward flexion), in which the last recorded range of motion passed these thresholds was 84.9% in external rotation, 75.5% in abduction, and 75.4% in forward flexion. The percentages would most likely be higher after completed treatment as the last recorded measurement was generally taken at the beginning of the last month of use. Without the HIS device, it is reasonable to postulate that these patients had a high likelihood of undergoing additional surgery and would still struggle to regain the ability to perform activities of daily living.

## Demographics

In addition to range of motion data, patient records from 2014 to 2015 also included the age and sex of each patient. There were 953 patients whose sex was included in their records and 856 patients where both age and sex were available. The average age was 49.9 years old for males and 50.5 years old for females (*p* = 0.33). There were 463 males and 490 females. Table 3 compares ROM between males and females. There were no significant differences between males and females in any ROM measurement in any plane of motion. The average time between device delivery and the last recorded range of motion measurement was 69.3 days in males and 70.3 days in females (*p* = 0.46).

**Table 3:** Range of motion comparison by sex reported as mean (SD)

Demographics	Sex		
	Male	Female	*p* value
***n***—**external rotation**	424	436	
Initial external rotation (°)	30.5 (20.7)	29.8 (19.6)	0.61
Last recorded external (°)	58.4 (20.68)	57.0 (20.8)	0.32
External rotation gain (°)	27.9 (24.9)	27.2 (22.9)	0.66
***n***—**abduction**	331	382	
Initial abduction (°)	84.6 (31.3)	83.7 (32.5)	0.72
Last recorded abduction (°)	118.6 (32.1)	121.4 (30.9)	0.23
Abduction gain (°)	34.0 (35.0)	37.7 (34.4)	0.16
***n***—*forward flexion*	248	245	
Initial forward flexion (°)	106.6 (36.0)	107.1 (33.3)	0.88
Last recorded forward flexion (°)	137.4 (28.5)	136.0 (26.2)	0.57
Forward flexion gain (°)	30.8 (345)	28.9 (33.3)	0.78
***n***—*internal rotation*	121	126	
Initial internal rotation (°)	44.9 (18.8)	45.8 (21.9)	0.74
Last recorded internal (°)	55.1 (17.8)	58.7 (19.6)	0.13
Internal rotation gain (°)	10.2 (22.4)	12.9 (24.0)	0.31

Table 4 shows the breakdown of range of motion data by age for the 856 patients whose age was included in the records. Patients ranged in age from 16 to 75 years old. The ROM gains were similar across all ages under 60, and gains decreased with age after 60 for all planes of motion other than forward flexion. The only significant differences measured through one-way analysis of variance were in days between delivery and last recorded measurement in external rotation, last recorded abduction, abduction gain, and initial forward flexion.

**Table 4:** Range of motion data separated by age

	Age						*p* value
	< 30	30–39	40–49	50–59	60–69	≥ 70	
***n***—*external rotation*	35	76	255	346	132	12	
Initial external rotation (°)	30.2	34.1	28.1	29.7	32.4	36.5	0.13
Last recorded external rotation (°)	56.1	61.9	57.3	57.5	57.1	53.6	0.54
External rotation gain (°)	25.9	27.8	29.2	27.8	24.7	17.1	0.35
Days between delivery and last recorded measurement	66.0	72.3	66.5	73.3	63.9	82.0	0.04
***n***—**abduction**	26	49	197	266	100	9	
Initial abduction (°)	82.9	86.5	82.9	83.3	86.7	88.2	0.90
Last recorded abduction (°)	110.3	129.8	124.2	119.1	114.5	107.6	< 0.01
Abduction gain (°)	27.4	43.3	41.3	35.8	27.8	19.4	< 0.01
Days between delivery and last recorded measurement	65.5	72.6	65.2	72.5	64.2	82.1	0.12
***n***—**forward flexion**	14	39	138	167	70	5	
Initial forward flexion (°)	124.1	120.1	102.6	107.3	105.4	118.2	0.03
Last recorded forward flexion (°)	136.6	145.9	135.6	134.7	137.3	150.8	0.24
Forward flexion gain (°)	12.5	25.8	33.0	27.4	31.9	32.6	0.21
Days between delivery and last recorded measurement	54.4	73.3	65.7	73.4	70.9	97.2	0.06
***n***—**internal rotation**	7	23	74	94	40	7	
Initial internal rotation (°)	33.6	47.5	44.5	43.8	49.3	54.0	0.31
Last recorded internal rotation (°)	48.3	55.1	59.3	58.4	53.9	57.0	0.51
Internal rotation gain (°)	14.7	7.6	14.8	14.6	4.6	3.0	0.23
Days between delivery and last recorded measurement	53.3	75.9	61.6	74.5	68.6	90.0	0.06

*p* values are from one-way analysis of variance

## Discussion

In this study, patients with shoulder stiffness who were treated with the HIS device showed significant gains in ROM in all four planes of shoulder motion regardless of age, sex, or starting ROM. The relevance of these improvements in ROM can be best explained in terms of activities of daily living. When the average final measured ROM for external rotation, abduction, forward flexion, and internal rotation for the patients in this study are compared to the clinically documented ROM required for activities of daily living, patients achieved the necessary motion improvement to return to normal activities.

A study by Namdari et al. reported the average ranges of motion required to complete 10 activities of daily living [23]. These activities are listed in Table 5. The necessary motion to complete all 10 activities was reported to be 59° of external rotation, 128° of abduction, 121° of forward flexion, and 102° of internal rotation. In a separate study by Khadilkar et al., the required range of abduction and forward flexion to complete 5 activities of daily living (opening a tight jar; pushing a heavy door; changing an overhead bulb; washing one’s hair; washing one’s back) was reported to be 121° for abduction and 111° for flexion [24].

**Table 5:** Activities of daily living used to determine the required ranges of shoulder motion

Activity
1. Placing a can of soup on an overhead shelf without bending the elbow
2. Reaching the small of the back to tuck in a shirt
3. Washing the middle of the back/unhooking a bra
4. Washing the back of the opposite shoulder
5. Placing a hand behind the head with elbow held straight to the side
6. Combing hair
7. Placing a can of soup on a shelf at shoulder level without bending the elbow
8. Placing a 1 gallon container on a shelf at shoulder level without bending the elbow
9. Reaching a shelf above the head without bending the elbow
10. Placing a 1 gallon container on an overhead shelf without bending the elbow

On average, patients in this study treated with the HIS device had a final recorded external rotation measurement of 59.6°. This level of external rotation would allow patients to perform all activities of daily living described in the Namdari study. The 122.6° of abduction seen at the final measurement in the HIS patients would allow them to complete 9 out of 10 activities from the Namdari study and all 5 activities from the Khadilkar study. The average final recorded abduction of HIS patients in this study was 5° below the reported range needed for “placing hand behind head with elbow out to side” in the Namdari study. The abduction ROM would likely show the greatest improvement between the last recorded ROM and the final ROM at the end of treatment because the device is switched to abduction stretching after external rotation stretching is completed; therefore the final month would most likely be focused on abduction stretching. The abduction at the end of the treatment period would likely be closer to or greater than the 128° needed to complete this task. For forward flexion, the 138.8° of flexion achieved by the HIS patients would allow them to perform all activities from both studies. Internal rotation data were only provided for 2 activities in the Namdari study (tuck in shirt behind back and wash the middle of the back/unhook bra). The patients in this study are short of this reported range on average. However, the measurements in the Namdari study may not correlate to clinical internal rotation measurement. If the patients were unable to achieve the necessary internal rotation to complete a task, they would have to compensate by using other planes of motion or by using alternative means to complete these two tasks (i.e., using a handheld scrub brush).

The ROM necessary for activities of daily living reported in both the Namdari study and the Khadilkar study was measured in healthy individuals. The fact that the patients in this study who were diagnosed with adhesive capsulitis can reach these same motion thresholds is a significant finding. The ability to perform activities of daily living is an important factor in returning to work and in avoiding any permanent impairment that could lead to disability payments. Non-surgical treatment using the HIS device is a safer option than a manipulation under anesthesia, which has a re-intervention rate of 14% (subsequent arthroscopy or repeat manipulation), and can lead to rare but severe complications including humeral fracture, glenoid fracture, shoulder dislocation, brachial plexus injury, or intra-articular damage to the cartilage or rotator cuff [25].

Treatment of shoulder motion loss using the HIS device can also allow a physical therapist to focus on other modalities during clinic visits other than range of motion (i.e., muscle strengthening or functional activities). The treatment protocol for the device is based on the TERT protocol which states that motion gain in a stiff joint is directly proportional to the stretching time at its end ROM [26, 27]. The patient has the ability to stretch their shoulder to the end ROM multiple times per day at home rather than being limited to stretching during 2 or 3 sessions of PT per week. The ROM gains made during PT visits can be maintained through use of the HIS device in at-home sessions to prevent rework by the physical therapist. Through use of the device, the patient can also increase their self-management (i.e., home program) and decrease their frequency of physical therapy visits over time.

This study was not without limitations. The number of reviewed patients that had complete sets of ROM data was limited based on the availability of clinical records. The ROM measurements were taken at various locations which could lead to variability in the initial and final measurements. However, measurements for a single patient were from the same source, so ROM gains should be consistent. In addition, the intertester reliability of range of motion measurements in the shoulder for all planes reported in this study has been previously reported as moderate to excellent. In one study, intertester intraclass correlation (ICC) scores were reported to be between 0.84 and 0.90 for flexion, abduction, and external rotation [28]. Another study reported ICC scores between 0.74 and 0.93 for flexion, between 0.76 and 0.95 for external rotation, and between 0.62 and 0.87 for internal rotation [29]. The reported ROM gains were likely underestimated because the final measurements were generally recorded from paperwork filed at the beginning of the last re-certification period and the gains made by the patient during this last period would not be included. However, gains in ROM for all shoulder motion were still excellent. There is also a mixed cohort of patients, but the combined data show that the HIS device can effectively treat motion loss in the shoulder caused by adhesive capsulitis as well as the remaining variety of motion loss conditions.

## Conclusions

The HIS device was shown to be effective in treating patients with shoulder motion loss due to adhesive capsulitis as demonstrated by the significant ROM gains seen in all planes of motion in the shoulder. The ability for a patient to recover lost motion quickly without surgery and to successfully complete activities of daily living is of great value to both the patient’s quality of life and in related healthcare cost savings. We believe this high-intensity stretch device should be considered for use by patients who are at risk for a motion loss surgery.

## Data Availability

The datasets analyzed during the current study are not publicly available due to their ownership by a private company but are available from the corresponding author on reasonable request.

## Abbreviations

HIS: High-intensity stretch
ICC: Intraclass correlation coefficient
MUA: Manipulation under anesthesia
PT: Physical therapy
ROM: Range of motion
SLAP: Superior labral tear from anterior to posterior
TERT: Total end-range time
